# Medical Smart Textiles Based on Fiber Optic Technology: An Overview

**DOI:** 10.3390/jfb6020204

**Published:** 2015-04-13

**Authors:** Carlo Massaroni, Paola Saccomandi, Emiliano Schena

**Affiliations:** Center for Integrated Research, Università campus Bio-Medico, Alvaro del Portillo, 21, Rome 00128, Italy; E-Mails: c.massaroni@unicampus.it (C.M.); p.saccomandi@unicampus.it (P.S.)

**Keywords:** smart textiles, fiber optic sensors, fiber Bragg grating sensors, respiratory monitoring, macrobending sensors, hetero-core fiber optics, Magnetic Resonance Imaging, MR-compatibility

## Abstract

The growing interest in the development of smart textiles for medical applications is driven by the aim to increase the mobility of patients who need a continuous monitoring of such physiological parameters. At the same time, the use of fiber optic sensors (FOSs) is gaining large acceptance as an alternative to traditional electrical and mechanical sensors for the monitoring of thermal and mechanical parameters. The potential impact of FOSs is related to their good metrological properties, their small size and their flexibility, as well as to their immunity from electromagnetic field. Their main advantage is the possibility to use textile based on fiber optic in a magnetic resonance imaging environment, where standard electronic sensors cannot be employed. This last feature makes FOSs suitable for monitoring biological parameters (e.g., respiratory and heartbeat monitoring) during magnetic resonance procedures. Research interest in combining FOSs and textiles into a single structure to develop wearable sensors is rapidly growing. In this review we provide an overview of the state-of-the-art of textiles, which use FOSs for monitoring of mechanical parameters of physiological interest. In particular we briefly describe the working principle of FOSs employed in this field and their relevant advantages and disadvantages. Also reviewed are their applications for the monitoring of mechanical parameters of physiological interest.

## 1. Introduction

The growing interest in smart textiles for medical applications is driven by the aim to increase the mobility of patients who need a continuous monitoring of physiological parameters [1,2,3,4]. Smart textiles are able to interact with the environment; therefore they embed one or more sensors to monitor various mechanical, thermal and chemical parameters (e.g., strain, temperature, displacement, oxygen blood saturation) [5,6,7]. During the last decades the use of fiber optic-based sensors (FOSs) has been gaining acceptance in a large number of applications in the fields of civil engineering, the automotive industry and medicine [8,9,10], among others. These sensors allow the measurement of physical and chemical parameters employing a large number of working principles and configurations [4]. FOSs can be divided into intrinsic sensors, where the fiber optic constitutes the sensing element, and extrinsic ones, where the fiber optic is only used as a medium to transport light. There are a number of reviews and books that focus on the description of FOSs [11,12,13,14,15], hence it is not possible to describe all the applications and working principles of FOSs in one journal article.

FOSs have good metrological properties (*i.e.*, low zero drift and sensitivity drift, good accuracy and good sensitivity and large bandwidth), they offer the possibility to implement distributed sensors and they are immune to electromagnetic interferences. These features make FOSs an emerging solution for the monitoring of physiological parameters and more generally for applications in medicine [9]. In fact, thanks to the mentioned valuable characteristics, FOSs can compete with other traditional electrical and mechanical sensors, and in many fields of application the superiority of their performances has been demonstrated. Lastly, the possibility to develop Magnetic Resonance (MR)-compatible sensors further motivates the growing interest in this technology.

Fiber optic technology is particularly attractive for application in smart textiles because it allows both sensing and signal transmission. Moreover, polymer optical fibers (POFs) are cheap, lightweight, flexible and robust, and they are able to measure high strain values without damage.

During the last decade, several groups of research have focused their efforts on obtaining a substantial development in the integration of smart textiles and fiber optic technology [16,17] as reported in a recent review [18].

In this paper, we aim at describing the use of smart textiles in medicine employing FOSs; in particular we focus on their use for monitoring physiological parameters by measuring mechanical variables and on the metrological properties and performances of the devices reported in literature. Also reviewed are the working principles of FOSs most frequently used in smart textiles for the mentioned applications. In Section 2 the working principles of the FOSs used in smart textiles and their applications in medicine are described. In Section 3 the applications in physiological monitoring of smart textiles based on FOSs along with their main advantages and drawbacks are reviewed.

## 2. Working Principle of Fiber Optic Sensors Used in Smart Textiles

FOSs can be designed using a large number of working principles. In this section the ones used to develop smart textiles for measuring mechanical variables in physiological monitoring are reviewed. In particular we focus on sensors based on the fiber Bragg grating technology and on intensity-based FOSs. The next subsections describe the working principle of these sensors with a brief description of their application in medicine.

### 2.1. Fiber Bragg Grating Sensors

Fiber Bragg grating (FBG) sensors consist of a periodic perturbation of the refractive index along the fiber core length obtained by exposure of the core to an intense optical interference pattern. Hill reported the first fabrication of FBGs in the late 1970s [19], but a major breakthrough occurred a decade later, when Meltz *et al.* improved the technique for their fabrication [20]. This last study can be considered the milestone, which enabled the development of FBG sensors for a large number of applications.

**Figure 1:**
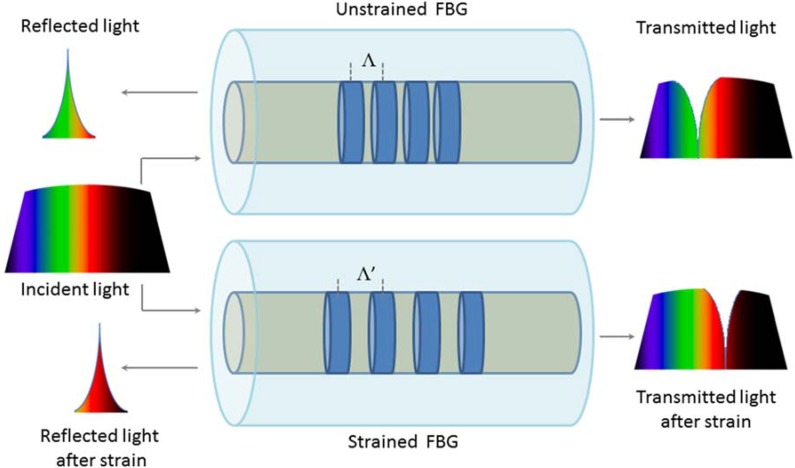
Schematic of the working principle of Fiber Bragg grating (FBG) sensors, and its response to strain.

Basically, an FBG can be considered as a short segment of a fiber optic (usually FBG longer than 3 mm–6 mm are employed, although for particular application smaller FBG are required), which reflects a narrow range of wavelengths and transmits all others. A schematic representation of the working principle of an FBG sensor and of its response to strain is shown in Figure 1.

The wavelength of the input light that is back-reflected (λ_B_) is sensitive to temperature and strain. In fact, λ_B_ can be expressed by the following equation, which is the first-order Bragg condition of the grating:

(1) $${\text{λ}}_{B}=2\cdot \text{Λ}\cdot {\text{η}}_{\text{eff}}$$

where Λ is the spatial period of the grating and η_eff_ is the effective refractive index of the fiber core. Both the terms are sensitive to strain and temperature, as a consequence, the use of a proper configuration allows estimating strain and temperature or both, by monitoring changes of λ_B_. The dependence of λ_B_ can be described by the fractional changes of Λ and η_eff_:

(2) $$\frac{\Delta {\text{λ}}_{B}}{{\text{λ}}_{B}}=\frac{\Delta \text{Λ}}{\text{Λ}}+\frac{\Delta {\text{η}}_{\text{eff}}}{{\text{η}}_{\text{eff}}}$$

Actually, due to the different sensitivity of Λ and η_eff_ to temperature changes and strain, the FBGs sensors used to estimate strain are based on the λ_B_ shift due to Λ changes, the FBG sensors used to monitor temperature are based on the λ_B_ shift due to η_eff_ changes. Equation (1) shows an important advantage of the FBG: their output is not affected by fluctuation of source intensity.

FBG sensors are employed in a large number of industrial fields to monitor different physical variables, including, among others, temperature, pressure, flow and vibrations. Moreover, FBGs have been largely used to monitor physiological parameters and more in general in medical fields, such as stroke volume, blood pressure and heartbeats [21,22], in microsurgery [23,24], foot pressure in diabetic patients [25], in biomechanical studies [26,27], in the monitoring of temperature during thermal ablation of cancer [28,29,30,31], in respiratory monitoring system [32,33], and in tactile sensing [34,35].

FBGs sensors are considered to hold great potential for application in medicine due to their good metrological properties. Moreover their characteristics of biocompatibility, non-toxicity and chemical inertness, as well as their small size and flexibility make them particularly attractive for invasive measurements during *in vivo* trials [36]. Lastly, they are suitable for application in environments with high electromagnetic noise, thanks to their immunity to electromagnetic interferences and their intrinsic MR-compatibility [37].

### 2.2. Intensity Modulated FOSs

**Figure 2:**
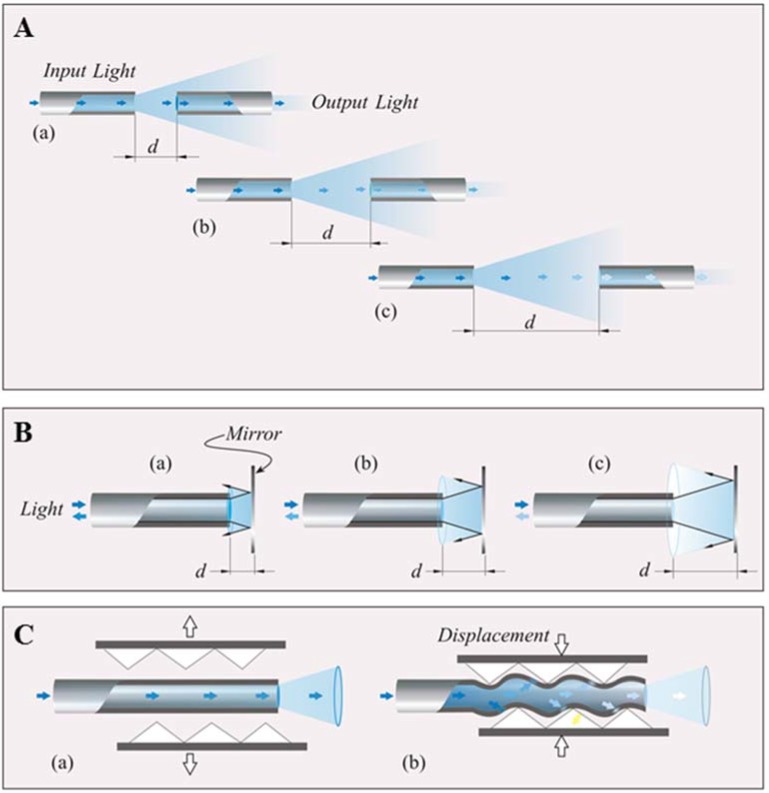
(**A**) Schematic of the working principle of an intensity modulated sensor using two fiber optic; (**B**) schematic of the working principle of an intensity modulated sensor using a fiber optic and a mirror; (**C**) schematic of the light lost from the fiber core caused by bending (adapted from [10]).

Intensity-modulated FOSs modulate light intensity, measured by a secondary element (e.g., a photodiode), in response to an environmental effect. A simple configuration of this kind of sensor is shown in Figure 2A. Two optical fibers are held in close proximity to each other; the light is injected into one of the optical fibers; as the light expands into a cone of light, its intensity, emitted by the first fiber and conveyed into the other one, depends on the distance (*d*) between the two fiber tips. Therefore, the light intensity can be considered an indirect measurement of the distance between the two fibers and of other physical variables influencing this distance. A similar configuration can be designed either by using a single fiber and a mirror (Figure 2B) or by using more than one fiber to obtain a differential configuration. The differential solution allows neglecting the influence of the light source intensity on sensor output. Another configuration to develop intensity-modulated FOSs is underpinned by the phenomenon that the light is lost from an optical fiber when it is bent. In particular a bent radius causes a leakage of the light traveling within the core of the fiber into the cladding with a resulting intensity modulation of light propagating through an optical fiber (Figure 2C). Macrobending sensors based on hetero-core fibers have been proposed to measure several physical properties [38,39].

Intensity-modulated FOSs have been used in different medical fields starting from the late 1960s, when Lekholm and Lindstrom proposed a sensor for intravascular pressure monitoring [40]. A similar sensor was proposed to monitor the intracranial pressure [41], and commercially available intracranial pressure intensity-modulated FOSs are produced by Camino Laboratories Inc. Their performances are largely investigated in clinical settings [42]. Moreover these sensors are used for pressure and temperature monitoring [43].

Macrobending FOSs find applications in medicine mainly in the monitoring of respiratory movements [44,45,46]. These sensors are largely used in smart textiles, therefore their medical applications will be described in more detail in the following section. A particular approach based on the bending of the fiber is also used to develop flow sensors for mechanical ventilation [47,48].

## 3. Smart Textiles Based on Fiber Optic Sensors: Medical Applications

Smart technical textiles are by definition textiles that can interact with their environment. Their ability to sense physical and chemical parameters can be accessed by embedding several kinds of sensors. The use of FOSs on textile is particularly attractive in some medical fields because of the possibility to use this technology during MR procedures, the cost reduction of key optical components and the improvement of the component quality, as well as the good metrological properties of these sensors. In particular, polymer optical fibers (POFs) match well with the requirements for application in smart textiles, being cost effective, lightweight and robust; moreover POFs are able to measure high strain values of several ten percent without fiber damage [49].

Significant developments of the integration of FOSs in textiles were driven by several groups of research, and in particular by the group involved in the European project OFSETH (optical fiber sensors embedded into technical textile for healthcare). OFSETH aimed at investigating the possibility to use FOSs embedded into textiles to monitor several physiological parameters.

In the following two subsections the application of smart textiles based on FBG sensors and on intensity modulated FOSs are described.

### 3.1. Smart Textiles Based on FBG Sensors: Medical Applications

FBG is one of the most frequently employed technologies to design smart textiles based on fiber optics, thanks to their good sensitivity to strain. This characteristic allows developing several configurations based on the measurement of the strain experienced by the FBG sensor to monitor different parameters of physiological interest.

**Figure 3:**
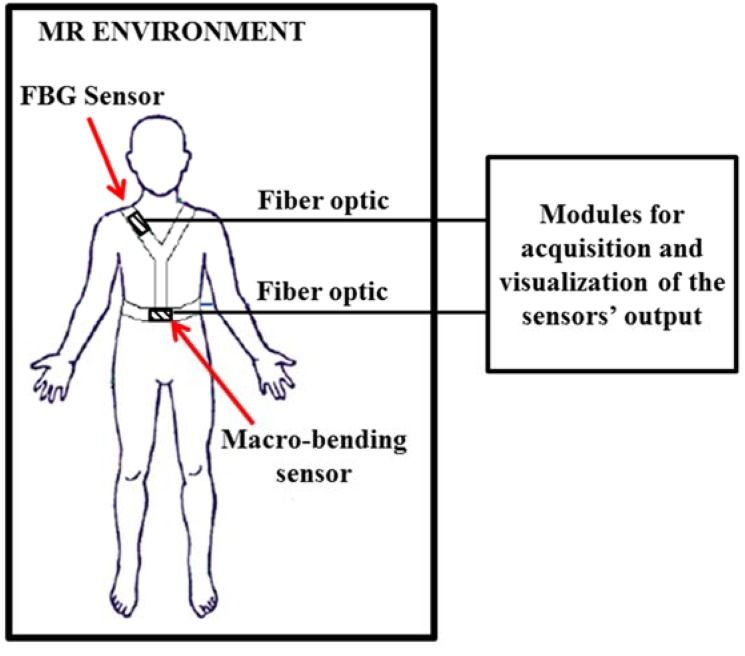
Schematic representation of the monitoring systems proposed by and developed in the optical fiber sensors embedded into technical textile for healthcare (OFSETH) project.

In particular this solution has been employed to monitor respiratory movements. The use of FBG to monitor respiratory movements and breathing rate has been demonstrated in the past [32,50,51], but only during the last decade have they been embedded in smart textiles. A number of studies regarding this topic have been proposed by the groups involved in the OFSETH project. A smart textile embedding two different FOSs (*i.e.*, FBG and macrobending) for respiratory monitoring has been developed. FOSs and the use of MR-compatible connectors allow the use of the proposed smart textiles on anesthetized patients during MR procedures. Indeed, these sensors are free from metallic or electrical conductive wires (when using a custom made MR-compatible connector); in addition, they are remotely interrogated via an optical fiber cable allowing the location of the monitoring unit outside of the MR field [52,53], as schematically reported in Figure 3.

**Figure 4:**
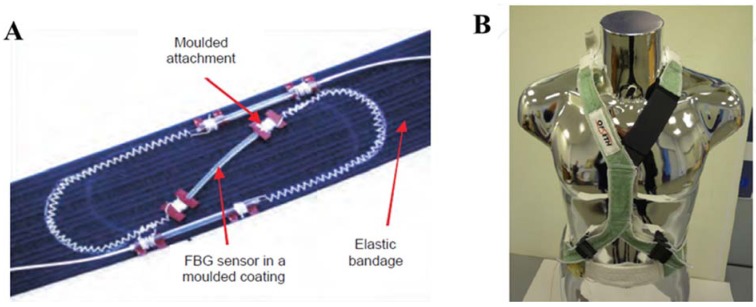
(**A**) Design of the FBG sensor developed in OFSETH project (adapted from [57]); (**B**) MRI-compatible sensing harness which embeds the fiber optic sensors for respiratory monitoring (adapted from [57]).

The high FBG sensitivity to strain (*i.e.*, ≈1.2 pm·µε^−1^) allows discriminating small strains; therefore they have been used to monitor thoracic movements that are smaller than abdominal ones. Two different methods to embed the FBG sensors within textiles (stitching and crochet) have been proposed. The calibration of the system, which embeds the FBG by stitching (see Figure 4A), has been performed by stretching the textiles in steps of 0.4% up to 40%. During the calibration, the FBG experiences strains up to 0.8%. The system shows good linearity from about 8.5% to 35%–40% of textile stretching, with a sensitivity of 0.35 nm/% and an accuracy better than 0.1% of elongation. Only preliminary experiments on the textiles developed with the crochet method have been performed. The integration of this sensor with a different FOS has been assessed on ten healthy volunteers [54]. Trials to evaluate the long-term properties and the stability of the respiratory sensor by an *ad hoc* developed simulator have been performed as well [55]. In particular the mechanical robustness of the sensor has been tested with more than 90,000 cycles in 129 hours with a simulated breathing rate between 10 breaths per minute and 12 breaths per minute [56]. A further valuable characteristic of the proposed system (see Figure 4B) is that it enables the continuous measurement of respiratory movement providing free access to all vital organs for medical staff actions [57].

A recent interesting study proposed a simple wearable system based on a single FBG sensor, which allows the simultaneous detection of both heart rate and respiratory cycles [58]. The main innovation is related to the structure in which the FBG sensor is embedded, which is [9] a PVC laminate resulting in a strain-sensitive foil, manufactured by an industrial spread-coating process. This integrated solution shows a sensitivity of 0.8 pm·µε^−1^. The authors performed trials on healthy volunteers, using two filters for breathing rate monitoring (band-pass filter tuned in the range 0.1–0.4 Hz) and cardiac frequency monitoring (band-pass in the range 0.5–1.3 Hz). The same group of research developed a system for breathing rate and cardiac frequency monitoring able to work in a wide range of temperature [59]. The authors selected the polychloroethanediyl as carrier material due to its excellent performance/cost and to the good sensitivity to strain. The system showed a linear response for elongation ranging from 0.6% to 1.6%. In this range the FBG output experienced an increase of about 8 nm; therefore the sensitivity was about 8 nm/%. They also investigated the sensors’ output changes with changes in temperature and found an increase of Bragg wavelength of about 1.5 nm for a temperature increase of 140 °C (a thermal sensitivity of about 10.7 × 10^−3^ nm/°C).

Other simple solutions have been proposed to monitor respiration and heart activity. In particular, Dziuda and coauthors developed a system which consisted of a Polymethyl methacrylate (PMMA) board with the size of 220 × 95 mm^2^ and a thickness of 1.5 mm. An FBG sensor was attached with epoxy adhesive [60]. They experimentally assessed the error of the system in the measurement of breaths per minute and heartbeats per minute during magnetic resonance imaging examinations, showing promising results (about one breath per minute and about three heartbeats per minute). The same group of researchers reported a system based on two FBG sensors positioned orthogonally to each other [61]. This group validated their system on three patients during MRI procedures by comparing their results with the ones obtained by an MRI-compatible portable module [62]. The results were promising (a relative error lower than 8% can be considered satisfactory considering that the system is intended for monitoring rather than diagnosis). Recently, they proposed a system for heart rate monitoring. It is MR-compatible and has been tested on seven volunteers showing a root mean square error of less than six beats per minute [63].

Allsop and coauthors developed a wearable system for respiratory function monitoring [64]. Their system employs an array of 40 in-line FBG sensors that produce 20 curvature sensors at different locations, each sensor consisting of two FBGs. They carried out experiments to measure the absolute volumetric changes of the human torso and estimated an error of 6% on the average volume.

Lastly, Li and coworkers developed a wearable sensor in intelligent clothing based on FBG for human body temperature monitoring [65]. They partly embedded an FBG in a polymer filled strip to improve the sensitivity of the measuring system. This way they obtained a temperature sensitivity of 150 pm·°C^−1^, almost 15 times higher than that of the bare FBG. To measure human body temperature, they distributed five FBGs in five places (*i.e.*, left chest, right chest, left armpit, right armpit and at center of the upper back). Moreover, they developed a model to estimate the body temperature by the data of the five FBGs. With this method they found an accuracy of ±0.1 °C.

The main characteristics of the abovementioned smart textiles and their applications are reported in Table 1.

**Table 1:** Smart textiles and wearable systems based on fiber optic sensors: working principle, medical application and metrological properties.

Reference	Working Principle	Medical Application	Metrological Properties and Other Features
[52,53,56]	Silica FBGs	Respiratory monitoring during MRI procedures	Non-invasive; MR compatible; good linearity in a wide range of strains with sensitivity = 0.35 nm/%; accuracy better than 0.1% of elongation
[58,59]	Silica FBGs	Cardiac and Respiratory monitoring during MRI procedures	Non-invasive; MR compatible; sensitivity of 0.8 nm/µε^−1^
[59]	Silica FBGs	Cardiac and Respiratory monitoring	Non-invasive; Sensitivity of 8 nm/%; good linear trend; thermal sensitivity ≈ 10.7 × 10^−3^ nm/°C
[60,62]	Silica FBGs	Cardiac and Respiratory monitoring during MRI procedures	Non-invasive; MR compatible; Simple design; Good accuracy in terms of breathing rate (±1 bpm) and heartbeat per minute (±3 bpm); relative error in patients during MRI procedures <8%
[61]	Silica FBGs	Cardiac and Respiratory monitoring during MRI	Non-invasive; Simple design; Flat frequency response in the range of interest (0.5 Hz up to 20 Hz); maximum relative error of 12%
[63]	Silica FBG	Heart rate monitoring	Non-invasive; MR compatible; Root mean square error lower than 6 beats per minute
[64]	Silica FBGs	Respiratory function monitoring	Non-invasive; 6% of error on the average volume
[65]	Bare FBG	Body temperature monitoring	Non-invasive; Sensitivity of 150 pm/°C in the range of interest (from 33 °C to 42 °C); accuracy 0.1 °C
[52,53,56]	Macro-bending/OTDR technique	Respiratory monitoring	Non-invasive; MR compatible; Good sensitivity stability after 172800 cycles (variations < 10%); low cost component for their interrogation
[66]	Intensity modulated	Respiratory monitoring	Non-invasive; MR compatible; low cost component
[67,68]	Intensity modulated	Respiratory monitoring	Non-invasive; low cost component
[69,70]	Macrobending hetero-core fiber optic	Respiratory monitoring	Non-invasive; low cost component; good agreement with the breathing rate measured by a commercial device
[71]	microbending	Respiratory monitoring during MRI procedures	Non-invasive; MR compatible; Accuracy better than ±2 breaths per minute
[72]	microbending	Respiratory rate and heart rate	Non-invasive; MR compatible; Accuracy better than ±2 breaths or beats per minute for respiratory monitoring heart rate
[73]	microbending	Heartbeat and respiratory monitoring	Non-invasive; low cost component; good agreement with the heart beat measured by a commercial device

### 3.2. Smart Textiles Based on Intensity-Modulated FOSs

FOSs based on intensity modulation and in particular macrobending sensors are employed to develop smart textiles for monitoring of physiological parameters.

**Figure 5:**
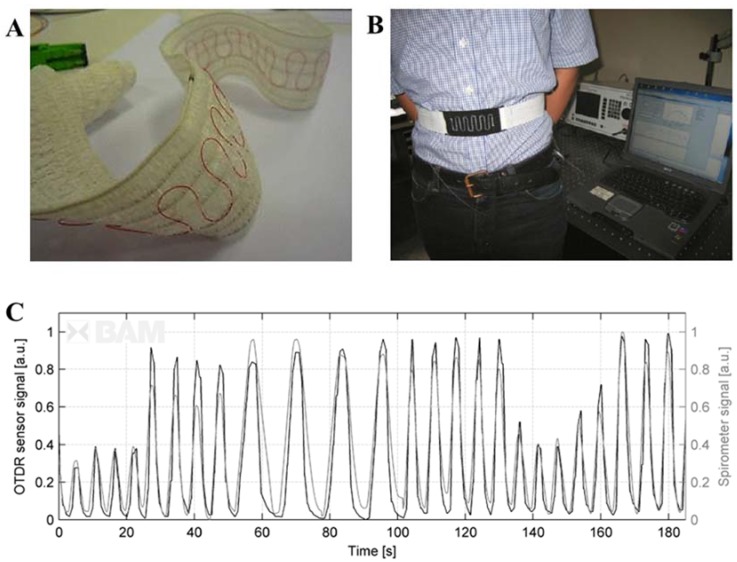
(**A**) Textiles based on macrobending FOS embedding a standard single mode fiber (adapted from [57]); (**B**) Macrobending sensors for monitoring of abdominal movements (adapted from [57]); (**C**) Output of the OTDR sensors during the monitoring of abdominal movement (adapted from [57]).

Textiles based on these kinds of FOSs were proposed by the groups of research involved in the OFSETH project. In particular, they developed smart textiles based on macrobending FOS for the monitoring of respiratory rate on patients during MRI procedures [52,53]. The sensors are MR-compatible and the monitoring devices are located out of the magnetic resonance environment (see Figure 3). A standard single mode fiber was embedded within a textile as shown in Figure 5A. This macrobending sensor, due to the lower sensitivity than the FBG sensors, was used to monitor abdominal movements, because these movements are larger than thoracic ones (Figure 5B). The authors found large oscillations in the sensor’s output during the stretching of the textile, which could cause a wrong computation of the breathing rate. As a consequence, they designed a sensor with more periods of the U-shape on the textile bandage (Figure 5B). This solution allowed to substantially increase the sensitivity (e.g., for a textile elongation of 38%, the sensor response increased from less than 3 dB with a single-loop design to more than 28 dB with a 10 loops-design). Lastly they developed a sensor based on the optical time-domain reflectometry (OTDR) in polymer optical fiber (POF). Basically, the macrobending entails a change of the backscattering in POF that can be sensed by the technique of OTDR [56]. This solution allows measuring strain in different locations of a single fiber (distributed measurement). A respiratory simulator was employed to test the robustness of both the macrobending sensor and the sensor based on the OTDR technique. Both these sensors show low variations after cycles at 10 breaths per minute and elongation up to 3% and 5% for the OTDR sensor and for the macrobending one, respectively. These sensors were also tested on healthy volunteers.

An interesting solution to develop an MR-compatible sensor wearable sensor for respiratory monitoring based on intensity modulation was presented in [66]. It consists of a PMMA tube, a mirror, a spring and a plastic optical fiber. Abdominal movement causes a variation of the distance between the mirror and the distal end of the plastic optical fiber that is related to the intensity of reflected light coupled to the fiber (as shown in the schematic representation in Figure 2B). The authors also tested the sensor during an MR procedure, and they did not find any negative effects related to patient safety and image quality.

Krehel and coauthors developed a textile for respiratory monitoring based on FOS previously described [67,68]. Basically, the working principle of the sensor employed can be explained as follows: the fiber optic geometry changes when a force (or pressure) is applied on the fiber; these geometry changes affect the wave guiding properties and hence induce light loss in the optical fiber. They characterized the sensor in [67], showing a range of measurement for force applied on 3 cm of fiber length up to 40 N, with a discrimination threshold of 0.05 N. They also performed the feasibility assessment of the wearable system for breathing rate monitoring [68]. The trials were performed at two breathing rates and at different positions of the sensing textile on the human body. Lastly, the comparison with a commercial device for respiratory measurements was performed using the Bland Altman analysis. The results showed that a large part of the differences between the measurements obtained by the FOS textile and the commercial device was concentrated in the range ±3 min^−1^, and the limits of agreement were about ±6 min^−1^.

Alemdar and coauthors developed a smart textile based on macrobending hetero-core fiber optic for respiratory movement analysis [69,70]. They embedded within a textile, a periodic macrobending hetero-core fiber optic that formed a periodic “U”. They experimentally assessed the increase of sensitivity to strain with the number of loops (ranging from one to seven loops) and the influence of the loop length on sensitivity. Then, the most sensitive configuration was tested to monitor the abdominal movement on a healthy volunteer.

Lau *et al.* designed a simple microbend fiber optic sensor for respiratory monitoring during MRI procedures [71]. They assessed the sensor feasibility during MRI procedures on twenty healthy volunteers, showing an accuracy of ±2 breaths per minute in the measurement of frequency rate. They also developed a system to monitor both respiratory rate and heart rate with a similar approach [72]. They tested the system on 11 volunteers during an MRI procedure, showing an accuracy of two breaths per minute for respiratory rate monitoring, and two beats per minute for heart rate monitoring.

Recently, a smart textile based on the use of a periodic fiber optic microbend sensor has been proposed for heartbeat and respiratory monitoring [73]. The authors integrated a section of multimode optical fiber sandwiched between parallel strips acting as a microbender onto an elastic substrate. Basically, the system monitors respiration and heartbeat by detecting the vibration caused by these actions. The microbending results in light loss, which is detected by a photodetector. After a set of *in vitro* experiments, they tested the textile on healthy volunteers. The measured heartbeat was compared to the one measured by a commercial device, the measured respiratory rate with the number of cycles manually counted in one minute.

The main characteristics of the abovementioned smart textiles and their applications are reported in Table 1.

## 4. Discussion

Smart textiles are used in a number of industrial fields such as civil engineering, transport and medicine, amongst others. Regarding the application in medicine and healthcare, they allow the continuous monitoring of physiological parameters of great importance (e.g., respiratory activity and heartbeat).

The monitoring of these parameters can be performed by several approaches, and different devices are commercially available. The main advantage to integrate FOSs into smart textiles is the possibility to develop MR-compatible systems. The number of MR scanners, the request for high field devices and for MR procedures in several medical branches (e.g., cardiology, surgery, orthopedics and neurology) are increasing worldwide. This trend reflects the growing need for MR-compatible systems able to monitor physical parameters inside the scanner, in order to provide real-time feedback about the status of the patient.

As a consequence, smart textiles based on the use of FOSs can be considered a potential new market niche in the field of healthcare monitoring. In this scenario is important to have a small size and lightweight system, and more in general a system allowing a normal range of motion and preserve the patient’s comfort. As a consequence, intensity-based fiber optic sensors seem to be indicated more than FBG ones that require the use of the interrogator. Moreover, also in homecare application the privacy and confidentiality of the patient must be respected. Solutions, such as enclosed rooms without traffic or others present and data transmission over secure lines can help to fulfill this requirement. Lastly, the problems of washability and, in some cases, sterilizability of FOS-based smart textiles are not well addressed. This feature must be considered because it can be a cost-effective option and can avoid the use of these systems for mono-patient or disposable applications. Therefore, future studies should address the robustness of these textiles against stress and washing cycles or their sterilizability The advantage is that FOSs are intrinsically safer than conventional sensors as they do not have any electrical connection with the patient and may be used at medium-term for monitoring different physiological parameters in order to replace standard sensors during MRI procedures. In this scenario it is also important to take into account the issue related to the connection of fibers. As reported by Kinet *et al.* [74] different solutions can be employed (e.g., commercially available standard connectors for optical fibers and *ad hoc* designed connectors). For the applications in the MRI environment, it is crucial to employ *ad hoc* designed, MR-compatible connectors.

Moreover, they can answer to the increasing demand for wearable systems for continuous monitoring of physiological parameters. A comfortable and wearable measuring system may significantly improve the quality of life of patients.

The main concerns related to the extensive use of smart textiles based on FOSs and their spread in commercially available products are related to the necessity to improve their stability due to light coupling (in particular in homecare long-term applications), and the abovementioned issue related to the connection of fibers (in particular in application during MRI procedures). The continuous improvement of the quality of the key optical components and their cost reduction, as well as the good metrological properties of these sensors motivates the increasing interest in this topic.

A recent review published by Quandt *et al.* in 2014 [18], described body-monitoring systems based on optical fibers and their application in the field of physiological monitoring. In this review, we devoted our attention especially to smart textiles based on FOSs for monitoring mechanical parameters of physiological interest. Also briefly reviewed are the working principles of the FOSs most used in these applications. Lastly, we focused on the metrological properties and on the performances of the systems reported in literature. In particular the most employed FOSs are based on FBG technology and on the modulation of intensity (in particular on macrobending). They are largely used for heartbeat and respiratory monitoring. These two kinds of sensors show complementary advantages and disadvantages in terms of sensitivity to movement and costs. FBG sensors are able to measure small strains thanks to their high sensitivity; on the other hand, in order to have good performances, interrogation must take place through an expensive device. The interrogation of intensity based FOSs is very simple and involves low cost and compact components, and their integration into textile fabrics may be very straightforward; on the other hand they are less sensitive than FBG sensors and are usually employed to monitor large movements (e.g., abdominal movements).

The use of smart textiles based on FOS in respiratory and heartbeat monitoring is just in its beginning stages, but their good metrological performances and the possibility to monitor patients in real time during MRI procedures motivates to continue research effort devoted to this topic.
